# Application of Cerium Dioxide Nanoparticles and Chromium-Resistant Bacteria Reduced Chromium Toxicity in Sunflower Plants

**DOI:** 10.3389/fpls.2022.876119

**Published:** 2022-05-04

**Authors:** Jing Ma, Huda Alshaya, Mohammad K. Okla, Yasmeen A. Alwasel, Fu Chen, Muhammad Adrees, Afzal Hussain, Salma Hameed, Munazzam Jawad Shahid

**Affiliations:** ^1^School of Public Administration, Hohai University, Nanjing, China; ^2^Cell and Molecular Biology Program, University of Arkansas, Fayetteville, NC, United States; ^3^Botany and Microbiology Department, College of Science, King Saud University, Riyadh, Saudi Arabia; ^4^Engineering Research Center of Ministry of Education for Mine Ecological Restoration, China University of Mining and Technology, Xuzhou, China; ^5^Department of Environmental Science and Engineering, Government College University Faisalabad, Faisalabad, Pakistan; ^6^Department of Environmental Sciences, The University of Lahore, Lahore, Pakistan; ^7^Department of Environmental Sciences, University of Jhang, Jhang, Pakistan

**Keywords:** bacteria, chromium, nanoparticles, phytoremediation, sunflower

## Abstract

The continuous increase in the heavy metals concentration in the soil due to anthropogenic activities has become a global issue. The chromium, especially hexavalent chromium, is highly toxic for living organisms due to high mobility, solubility, and carcinogenic properties. Considering the beneficial role of nanoparticles and bacteria in alleviating the metal stress in plants, a study was carried out to evaluate the role of cerium dioxide (CeO_2_) nanoparticles (NPs) and *Staphylococcus aureus* in alleviating the chromium toxicity in sunflower plants. Sunflower plants grown in chromium (Cr) contaminated soil (0, 25, and 50 mg kg^−1^) were treated with CeO_2_ nanoparticles (0, 25, and 50 mg L^−1^) and *S. aureus*. The application of Cerium Dioxide Nanoparticles (CeO_2_ NPs) significantly improved plant growth and biomass production, reduced oxidative stress, and enhanced the enzymatic activities in the sunflower plant grown under chromium stress. The application of *S. aureus* further enhanced the beneficial role of nanoparticles in alleviating metal-induced toxicity. The maximum improvement was noted in plants treated with both nanoparticles and *S. aureus*. The augmented application of CeO_2_ NPs (50 mg l^−1^) at Cr 50 mg kg^−1^ increased the *chl a* contents from 1.2 to 2.0, *chl b* contents 0.5 to 0.8 and mg g^−1^ FW, and decreased the leakage of the electrolyte from 121 to 104%. The findings proved that the application of CeO_2_ nanoparticles and *S. aureus* could significantly ameliorate the metal-induced stress in sunflower plants. The findings from this study can provide new horizons for research in the application of nanoparticles in phytoremediation and bioremediation.

## Introduction

Chromium is naturally found in the soil, air, and water. The trivalent chromium (Cr^+3^) and hexavalent chromium (Cr^+6^) are two forms of chromium (Yoshinaga et al., 2018). The hexavalent chromium is considered the most toxic form that mainly exits as chromate or dichromate. Human activities and industrialization have increased the addition of chromium in the environment, and it has become a significant concern for plants, animals, and humans due to its lethal effects. The primary source of chromium pollution is the leather industry, followed by the electroplating industry textile and chromate mining (Belay, 2010; Tumolo et al., 2020). Chromium is a toxic and non-essential element for plants, and plants do not have a specific mechanism for its uptake (Shanker et al., 2005). Chromium is highly toxic for plants due to its detrimental effect on plants’ growth and development. The most common effect of chromium on plants is reduced leaf and root growth, reduced yield, inhibition of enzymatic activity, and mutagenesis (Singh et al., 2013).

Sunflower (*Helianthus annuus* L.) is one of the essential vegetable oil sources, with an annual production of about 9 million tonnes. The sunflower oil is highly healthy due to its high concentration of mono-saturated and poly-saturated fatty acid and high contents of vitamin E (Kaya et al., 2012). Sunflower has been widely studied due to its unique ability to uptake and accumulate various heavy metals (Alaboudi et al., 2018; Chen et al., 2020). Sunflower can uptake and accumulate metals such as Pb, Cd, and Cr in its roots and shoots from contaminated soil (Chen et al., 2020). Due to its high biomass, sunflower is recommended to remediation soil contaminated with heavy metals. However, the high concentration of chromium has a detrimental effect on sunflower productivity and enzymatic activity (Fozia et al., 2008).

Phytoremediation is a green technology in which plants remove toxic pollutants and heavy metals from contaminated soil and water. In this technology, the potential of plants is applied to uptake, accumulate, and degrade pollutants from the contaminated medium (Hauptvogl et al., 2019). The ability of plants to remediate the pollutants depends upon the type of pollutants, concentration of pollutant and plant biomass production, and ability to accumulate metals (Ashraf et al., 2019). Hyperaccumulator plants have tremendous ability to accumulate particular metals and metalloids, hundred or thousand times greater than typical plants in the same environmental condition (Peer et al., 2005). These plants are most tolerant to high concentrations of heavy metals, can uptake these pollutants through their roots system, and accumulate high levels of these metals in their living tissue such as roots, stems, and leaves (Peer et al., 2005; Baker et al., 2020). Plants also have different metabolic processes to alleviate the metal stress, such as producing organic acids that can chelate the heavy metals and covert these metals into not toxic form and prevent the uptake of heavy metal by plants (Shahid et al., 2017, 2019). Other methods used by the plant to minimize the metal toxification include extracellular complexation and cytoplasmic complexation. The metal stress in the plant causes severe damage to the plant’s metabolic process. To tolerate the metals stress, the plant should activate the antioxidant enzymes system and repair the oxidative stress and damage caused by metal stress (Sharma et al., 2016).

The microorganism can be applied as a biological tool to remove the heavy metals from the polluted medium due to their ability to concentrate and recover heavy metals (Devi et al., 2021). Bioremediation is the application of microorganisms to remediate polluted sites contaminated with heavy metals and other organic/inorganic pollutants (Shahid et al., 2020). Bacteria have evolved efficient mechanisms to bio-remediate heavy metals. These mechanisms include detoxification, adsorption, oxidation, and reduction of heavy metals (Kang et al., 2016; Naik and Kumar, 2020). The versatility of bacteria to degrade various pollutants and heavy metals has emphasized their application in the remediation of different types of soils. The bioremediation has shown promising results in the remediation of heavy metals such as lead, chromium, and cadmium, even in deficient concentrations in soil and water where other processes failed (Tiquia-Arashiro, 2018; Pushkar et al., 2021). The phytoremediation process can be boosted by applying selective bacteria resistant to specific metals. It has been widely proven that the application of bacteria in phytoremediation increases heavy metals such as lead, chromium, and iron uptake and removal from the contaminated soils (Kong et al., 2019; Ma et al., 2019).

Nanotechnology has grown remarkably in the last two decades with its wide application in agriculture and environmental remediation (Song et al., 2019; Zand et al., 2020). Nanoparticle-assisted phytoremediation has emerged as a reliable technology for removing contaminated soil with metals and metalloids. The nanoparticle has been widely applied from remediation of lead, cadmium, and arsenic from contaminated soil (Song et al., 2019; Zhu et al., 2019). The nanoparticle may improve the phytoremediation by a direct effect such as directly removing the pollutant from the soil by adsorption and immobilizing the metals, and ultimately reducing the concentration of metals in the soil (Omara et al., 2019). The nanoparticle can also improve phytoremediation by promoting plant growth, such as carbon nanotubes, Ag nanoparticles, and ZnO nanoparticles (Sabir et al., 2014). The nanoparticle may also increase the plant tolerance to abiotic stress by regulating the gene expression of enzymes, such as application of silicon nanoparticles improved the phytoextraction capacity of pea plant for chromium (VI; Tripathi et al., 2015). The applied nanoparticle may also improve plant growth by increasing the availability and absorption of nutrients and water (Kale and Gawade, 2016).

Considering the influential role of nanoparticles and bacteria, a field study was conducted to analyze the potential of Cerium Dioxide Nanoparticles (CeO_2_ NPs) and a chromium-resistant bacteria *Staphylococcus aureus* in remediating the chromium from contaminated soil through sunflower plants. It was assumed that combined application of CeO_2_ NPs and chromium-resistant bacteria will alleviate the chromium toxicity by their mutual role and enhance plant tolerance to metal toxicity. The augmented and individual role of CeO_2_ NPs and *S. aureus* on the performance of chromium-stressed plants was evaluated by analyzing the changes in growth parameters, enzymatic activity, and oxidative stress. The finding from this research will provide valuable information about the application of nanoparticles and bacteria in remediating heavy metals from the contaminated medium.

## Materials and Methods

### Soil Sampling and Analysis

The soil for this experiment was collected from the field of Govt. College University, Faisalabad from various points and depths from 0 to 20 cm to make a homogeneous mixture as recommended by previous researchers (Rehman et al., 2018; Rizwan et al., 2019). The collected soil sample was stored at 4°C in cooler box to protect from sunlight. All collected soil samples were air-dried, and the plant debris and large soil particles were removed by sieving the soil through a 2 mm sieve. The pH of the soil was analyzed by making the soil water paste and noted by calibrated pH meter. Similarly, the electrical conductivity (EC) of the soil was noted by calibrated EC meter. The collected soil was analyzed for metal contents by treating ammonium bicarbonate diethylene triamine penta acetic acid (AB-DTPA) at pH 6.7 (Soltanpour, 1991). The soil was artificially spiked with K_2_Cr_2_O_7_ to make the chromium-contaminated soil just like the natural agricultural soil of district Qasur and Sialkot highly contaminated with chromium due to irrigation with tannery effluent.

### Bacterial Inoculation of Seeds

The soil was collected, ground, and sieved through a 2 mm sieve after drying at 70°C. The sieved soil was autoclaved at 121°C for 20 min to remove bacterial contamination. Nutrient broth and Cr-resistant bacteria, *S. aureus*, were used to make the bacterial inoculum. The bacterial inoculum was shaken at 2000 rpm for 48 h at 30°C, then centrifuged for 10 min at 6000 rpm. The supernatant was collected and diluted with distilled water (Zulaika and Sembiring, 2014). The density of the bacterial isolate was measured using a hemocytometer. The population size of the centrifuged bacterial cells was set at 2.8 × 10^8^ wet weight. Sunflower seeds were disinfected by inoculating them with a 10% sugar solution. The seeds were adequately coated with clay and an equal amount of peat moss (1:1) and placed overnight.

### Sunflower Sowing and Harvesting

The experiment was performed in the pots at the botanical garden of Government College University, Faisalabad, at temperatures 20–25°C with 70% humidity. Each plastic container was filled with 5 kg of sieved soil mixed with three chromium concentrations (0, 25, and 50 mg kg^−1^). Six uniform healthy sunflower seeds were rinsed with hydrogen peroxide solution (15% v/v) followed by tap and distilled water. Six sunflowers’ seeds are sown in each pot, and after germination, only three healthy and uniform seedlings were kept to grow for further proceedings. A mixture of nitrogen, phosphorus, and potassium was applied at the rate of 120:50:25 kg ha^−1^ for the healthy growth of the plants. After 2 weeks of germination, the nanoparticle CeO_2_ NPs were applied as a foliar spray in three concentrations (0, 25, and 50 mg L-1), while controls were treated with distilled water. Cerium (IV) oxide nano-powder (CeO_2_-NPs) was of Alfa Aesar with size, purity, and surface area of 15–30 nm, 99.5%, and 30–50 m^2^/g, respectively. The experiment was run for 4 months and when plants reached at maturity, the harvesting was done. The plants were harvested about 1 cm high from the soil, and plants were separated into different parts such as root, shoot, and leaves for further analysis. Fresh plant’s samples were kept in a cooler box during transportation to the laboratory for analysis to prevent changes in biochemical properties. The plants shoot and roots were appropriately washed with distilled water and roots were washed with 1% HCl followed by filtered water to remove the soil, contamination, and acid. The samples were air-dried and then oven dried at 72°C for 72 h.

### Plant Growth Parameter and Chlorophyll Contents

After harvesting, the plant height (cm) was measured by measuring tape; then, the shoot dry weight (g pot^−1^) and roots dry weight (g pot^−1^) were measured by weight balance. The number of leaves per plant was counted, and leaf area (cm^2^) was measured. The chlorophyll a, b, total chlorophyll, and carotenoid contents were measured by extracting 0.2 g of fresh leaves in 0.5 ml of acetone (3% v/v). The supernatant was obtained through centrifugation for 10 min at 10,000 rpm, and absorption was noted through spectrophotometer for chlorophyll a (*chl a*) at 663 nm, chlorophyll b (*chl b*) 645 nm, and carotenoid at 470 nm (Lichtenthaler, 1987; Gohari et al., 2020).

### Estimation of Antioxidant Enzymes and Reactive Oxygen Species Contents

The antioxidant activities of the enzymes (CAT, APX, POD, and SOD) in sunflower plants were determined using methods developed by Aebi (1984) for catalase (CAT), Nakano and Physiology (1981) for ascorbate oxidase (APX), and Zhang (1992) for peroxidases (POD) and superoxide dismutase (SOD; Nakano and Physiology, 1981; Aebi, 1984; Zhang, 1992).

The reactive oxygen species (ROS) were measured by analyzing the electrolyte leakage (EL), malondialdehyde (MDA) contents, and H_2_O_2_ in sunflower plants. The initial EC1 from the leaves was checked by extracting the supernatant by autoclaving the plants at 32°C for 2 h, then heating the sample at 121°C. The final EL contents were noted using the Dionisio-Sese and Tobita (1998) method (Dionisio-Sese and Tobita, 1998). The MDA and H_2_O_2_ contents were determined by preparing a supernatant solution by crushing a 0.1 g plant sample in liquid nitrogen and then in a 0.05 M phosphate buffer. This supernatant solution was prepared with TCA (0.1%) and TBA (0.5%) for MDA content, and absorbance was measured at 532 nm (Zhang and Kirkham, 1994). The H_2_O_2_ content was determined by mixing the supernatant with phosphate buffer and measuring the absorbance at 410 nm (Jana and Choudhuri, 1981).

### Metal Contents in Shoot and Roots

The concentration of Cr^+6^ and Cr^+3^ in plants roots and shoots sample was measured by acid digestion of root and shoot samples by the standard method (Apha, 2012). The roots and shoot samples of 1 g each were ground and acid digested using HNO_3_ and HCLO_4_ (3:1) ratio. The metals contents in digested samples were determined by atomic absorption spectrophotometer.

### Statistical Analysis

The data from the experimental research work are expressed as a mean value with a standard deviation. The replicated data were statistically analyzed using two-way variance (ANOVA) with Statistix 10.0 version software to recognize significant variance, and means were compared using Tukey’s post-hoc test. At *p < 0.05*, values were considered significant for all treatments.

## Results

### Effect on Growth Parameters

**Figure 1:**
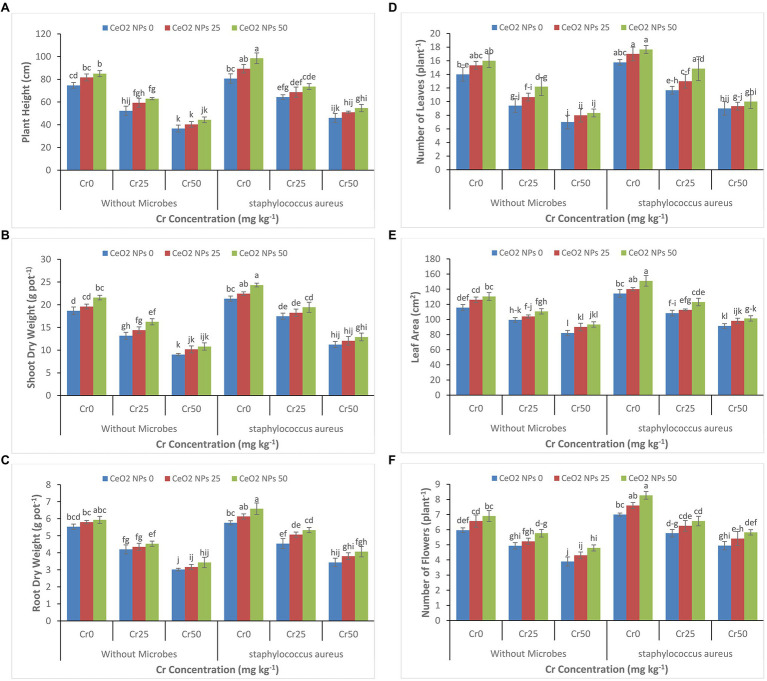
**(A–F)**: Effect of CeO_2_ NPs and *Staphylococcus aureus* on plant height **(A)**, shoot dry weight **(B)**, root dry weight **(C)**, number of leaves **(D)**, leaf area **(E)**, and number of flowers **(F)** in sunflower plant under Cr stress. The given values are means of three replications with standard deviation. The alphabets represent the significant/non-significant difference at *p* ≤ 0.05.

It is evident from Figures 1A–F that increasing the concentration of chromium from 0 to 50 mg kg^−1^ significantly decreased all plant growth parameters (plant height, shoot dry weight, root dry weight, number of leaves per plant, leaf area, and number of flowers per plant). At the highest concentration of chromium (Cr 50), the plant height decreased from 75 to 37 cm, and shoot dry weight decreased 19 to 9 g pot^−1^. A similar reduction was observed at a concentration of Cr50 for other growth attributes. However, *S. aureus* and CeO_2_ NPs significantly improved all plant growth parameters in sunflower plants under chromium stress. In plants with stress Cr50, the treatment with *S. aureus* increased the plant height up to 47 cm as compared to 37 in treatment without bacterial application. On the other hand, the application of CeO_2_ NPs further boosted the role of bacteria in plants under chromium stress. At stress level Cr50, the combined application of *S. aureus* and CeO_2_ NPs improved the plant height from 46 to 55 cm and shoot dry weight 11 to 13 g pot^−1^.

### Effect on Photosynthetic Parameters

**Figure 2:**
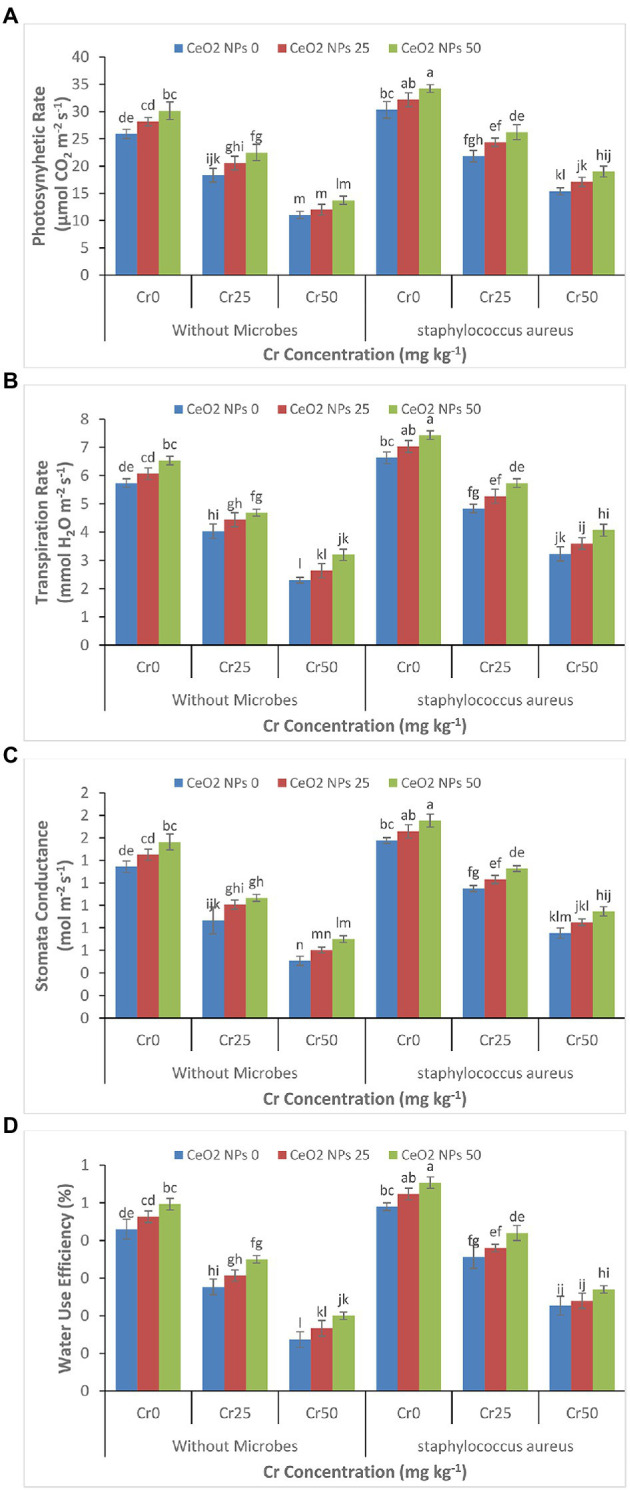
**(A–D)**: Effect of CeO_2_ NPs and *Staphylococcus aureus* on photosynthetic rate **(A)**, transpiration rate **(B)**, stomatal conductance **(C)**, and water use efficiency **(D)** in sunflower plant under Cr stress. The given values are means of three replications with standard deviation. The alphabets represent the significant/non-significant difference at *p* ≤ 0.05.

Just like the effect of chromium stress on growth parameters, the high concentration of chromium severely reduced the photosynthetic rate, transpiration rate, stomatal conductance, and water use efficiency in sunflower plants (Figures 2A–D). The increasing chromium concentration from 0 to 50 mg kg^−1^ reduced the photosynthetic rate from 25.9 to 11.0 μmol CO_2_ m^−2^ s^−1^ and water use efficiency from 0.4 to 0.1%. The application of *S. aureus* and CeO_2_ NPs significantly alleviated the damage to photosynthetic parameters by increasing the photosynthetic rate from 15.4 to 19.0 μmol CO_2_ m^−2^ s^−1^ and water use efficiency from 0.2 to 0.3% with increasing concentration of CeO_2_ NPs from 0 to 50 mg L^−1^.

### Effect on Chlorophyll Contents

**Figure 3:**
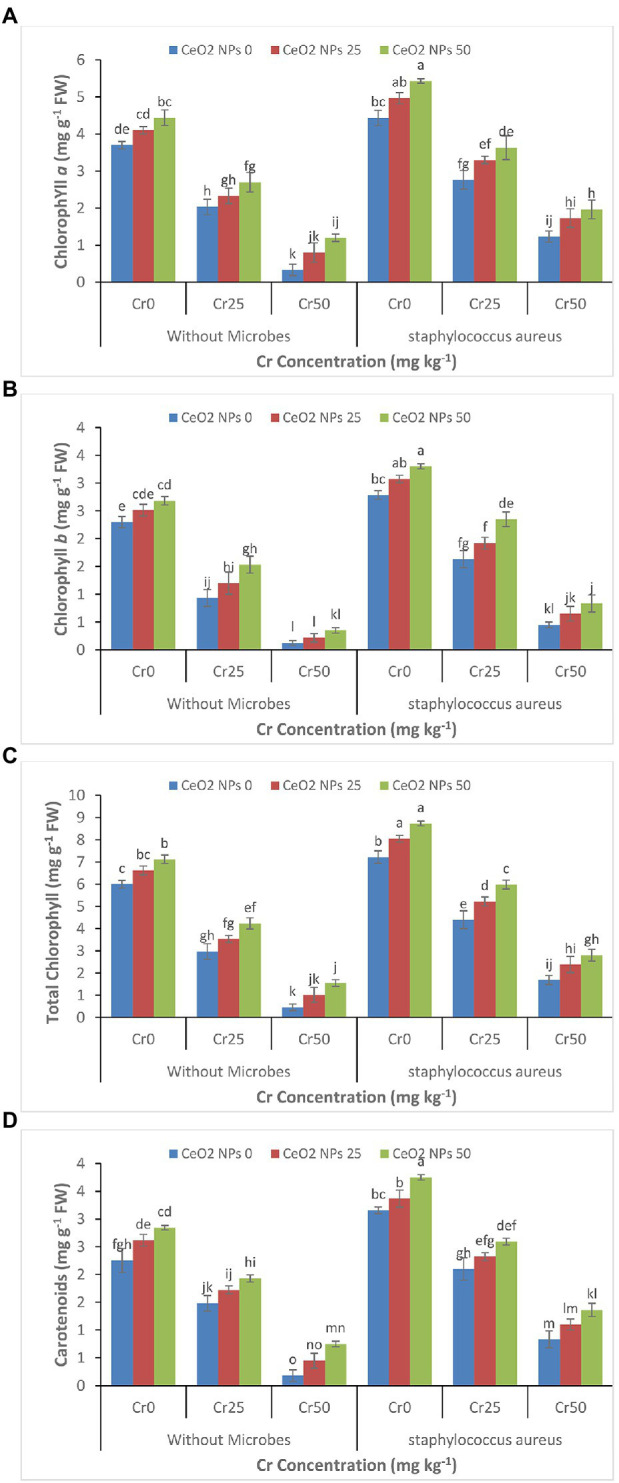
**(A–D)**: Effect of CeO_2_ NPs and *Staphylococcus aureus* on chlorophyll *a* **(A)**, chlorophyll *b* **(B)**, total chlorophyll **(C)**, and carotenoid content **(D)** in sunflower plant under Cr stress. The given values are means of three replications with standard deviation. The alphabets represent the significant/non-significant difference at *p* ≤ 0.05.

Under Cr stress, chlorophyll contents such as Chlorophyll a, chlorophyll b, total chlorophyll, and carotenoids continued to decrease gradually with increasing concentration of chromium (Figures 3A–D). Whereas, the successful application of CeO_2_ NPs and *S. aureus* individually and collectively enhanced the chlorophyll contents in the sunflower plants facing chromium stress. The maximum increase was observed by the combined application of CeO_2_ NPs and *S. aureus*. In plants under chromium stress Cr 50, without *S. aureus*, CeO_2_ NPs from 0 to 50 improved the *chl a* content from 0.3 to 1.2 mg g^−1^ FW. The combined application of *S. aureus* and CeO_2_ NPs from 0 to 50 improved the *chl a* contents from 1.2 to 2.0 mg g^−1^ FW.

### Effect on Enzymes Activity

**Figure 4:**
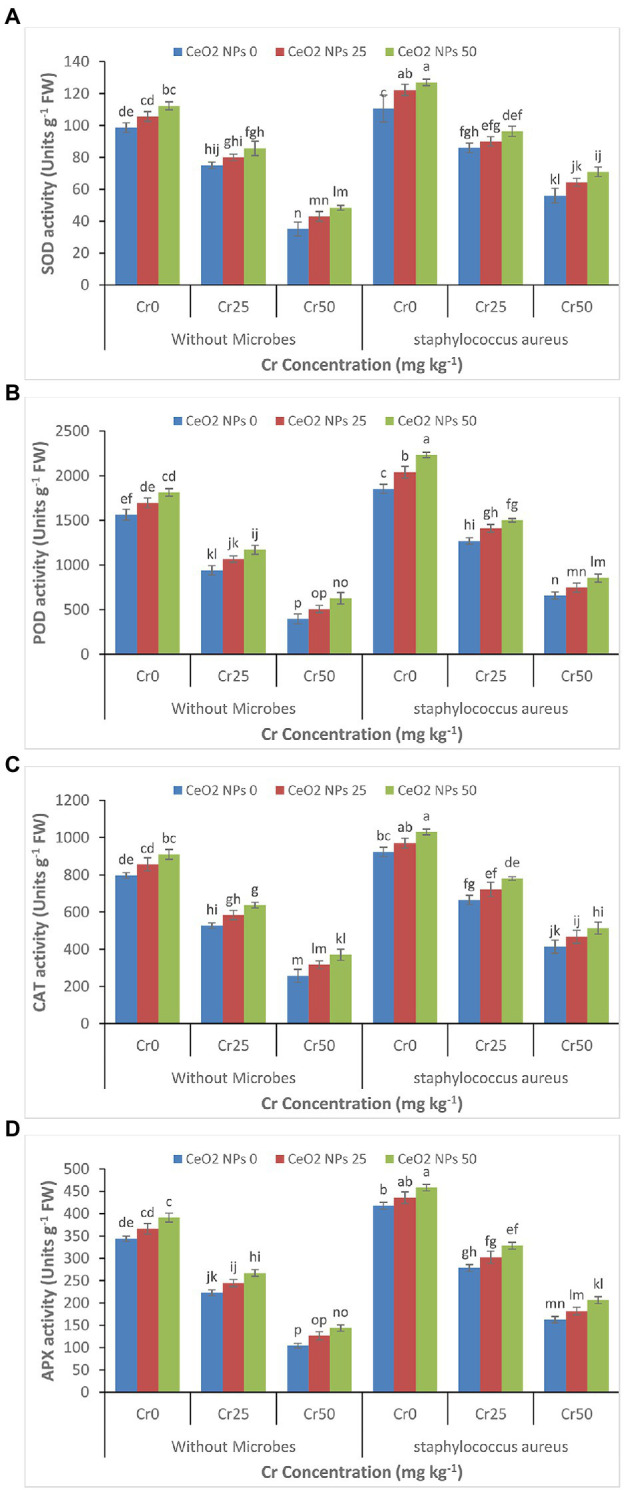
**(A–D)**: Effect of CeO_2_ NPs and *Staphylococcus aureus* on SOD activity **(A)**, peroxidases activity **(B)**, catalase activity **(C)**, and ascorbate oxidase activity **(D)** in sunflower plant under Cr stress. The given values are means of three replications with standard deviation. The alphabets represent the significant/non-significant difference at *p* ≤ 0.05.

The gradual decrease was depicted in enzymes activity (SOD, POD, CAT, and APX) in the sunflower plant with a gradual increase in the chromium concentration (0 to 50 mg kg^−1^; Figures 4A–D). The treatment of plants with CeO_2_ NPs alleviated the chromium stress and improved the plant’s enzyme activity under chromium stress. For instance, the SOD activity in plants under chromium stress Cr50 improved from 35 to 48 with increasing CeO_2_ NPs from 0 to 50 mg L^−1^. The maximum improvement in the enzyme’s activity in plants under the highest level of chromium stress (Cr50) was observed in plants treated with the combined application of *S. aureus* and CeO_2_ NPs. POD contents were increased from 657 to 853 Units g^−1^ FW.

### Effect on Oxidative Stress

**Figure 5:**
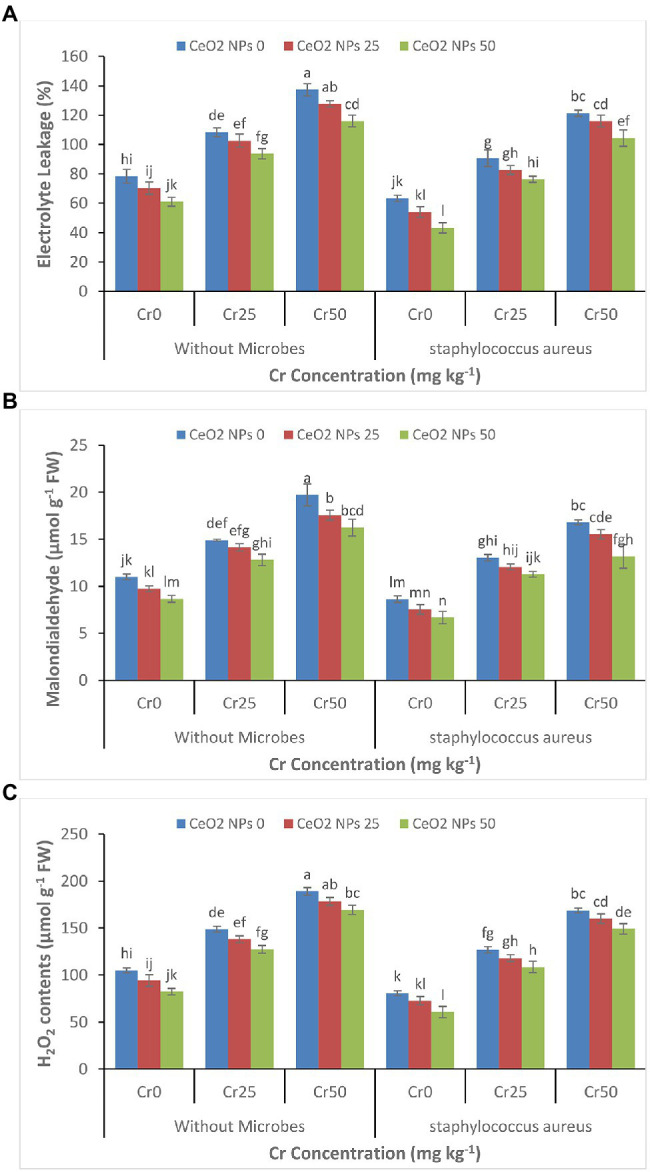
**(A–C)**: Effect of CeO_2_ NPs and *Staphylococcus aureus* on electrolyte leakage **(A)**, malondialdehyde contents **(B)** and H_2_O_2_ contents in sunflower plant under Cr stress. The given values are means of three replications with standard deviation. The alphabets represent the significant/non-significant difference at *p* ≤ 0.05.

The increasing concentration of chromium increased the oxidative stress, which was evident from the increasing level of electrolyte leakage, malondialdehyde contents, and H_2_O_2_ contents in the sunflower plants (Figures 5A–C). The increasing chromium concentration from 0 to 50 increased the EL contents by 78 to 137%, MDA contents of 11 to 20 μmol g^−1^ FW, and H_2_O_2_ contents of 105 to 189 μmol g^−1^ FW in treatments without application of *S. aureus* and CeO_2_ NPs. In contrast, CeO_2_ NPs significantly decreased oxidative stress by reducing the EL contents 137 to 116% in treatments with chromium stress Cr50 and without *S. aureus*.

### Effect on Chromium Uptake and Accumulation

**Figure 6:**
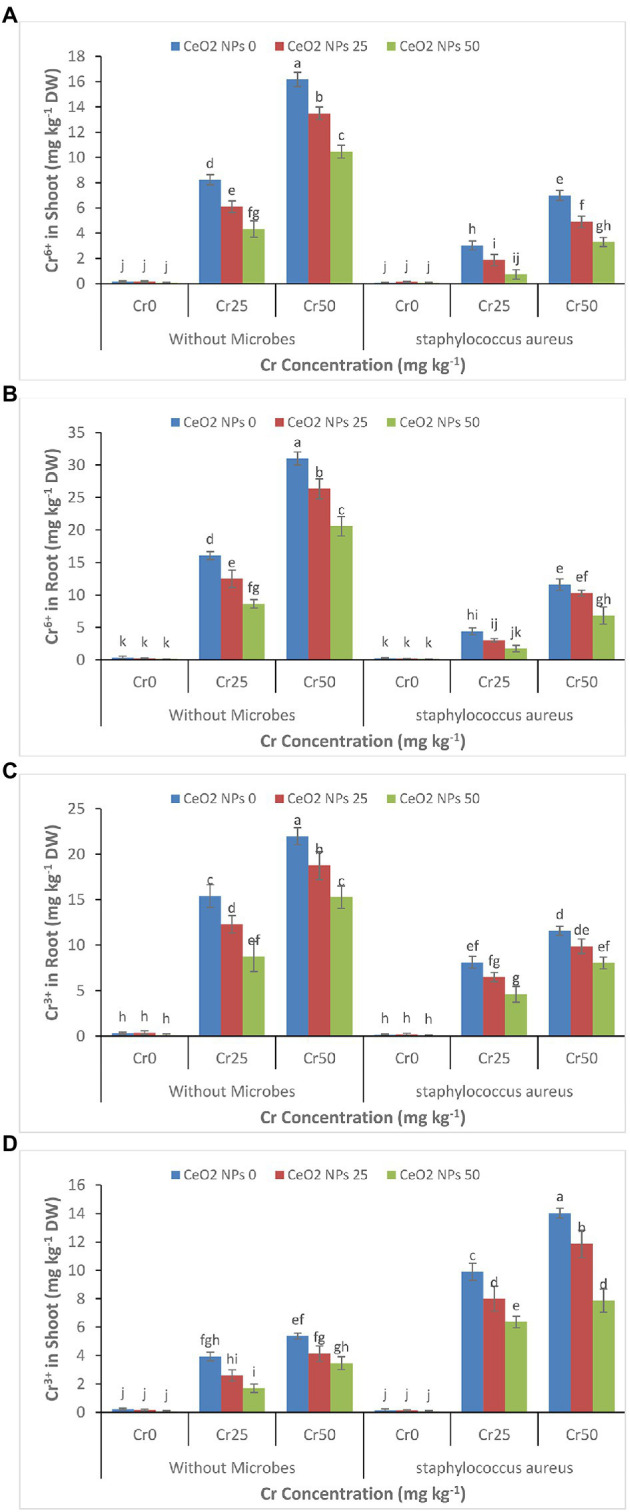
**(A–D)**: Effect of CeO_2_ NPs and *Staphylococcus aureus* on bioaccumulation of Cr^+6^ in the shoot **(A)**, Cr^+6^ in roots **(B)**, Cr^+3^ in the shoot **(C)**, and Cr^+3^ in roots **(D)** of sunflower plant under Cr stress. The given values are means of three replications with standard deviation. The alphabets represent the significant/non-significant difference at *p* ≤ 0.05.

The increasing concentration of chromium (0–50 mg kg^−1^) increased the accumulation of chromium Cr^+6^ and Cr^+3^ in plant roots and shoots (Figures 6A–D). The Cr^+6^ concentration increased from 0.4 to 31 and 0.2 to 16.2 mg kg^−1^ DW in the plant’s roots and shoots. A similar trend was observed for Cr^+3^ in treatments without the application of *S. aureus* and CeO_2_ NPs. The application of CeO_2_ NPs decreased the accumulation of Cr^+6^ and Cr^+3^ in plant root, and shoots such as Cr^+6^ concentration in plant root decreased from 31 to 20.6 mg kg^−1^ DW and Cr^+3^ concentration decreased from 22 to 15.3 mg kg^−1^ DW in plant under stress level Cr50. However, the application of *S. aureus* brought a dramatic change in the accumulation of chromium by facilitating the more accumulation of Cr^+3^ than Cr^+6^ in plant roots and shoot. In plants treated with *S. aureus*, the Cr^+3^ accumulation in roots was 8.0 mg kg^−1^ FW compared to Cr^+6^ concentration, which was 6.8 mg kg^−1^ FW and 7.9 Cr^+3^ as compared to 3.3 mg kg^−1^ DW Cr^+6^ in the shoot at stress level Cr50.

## Discussion

In this study, for the first time, we showed that the application of CeO_2_ NPs and bacteria *S. aureus* could alleviate chromium-induced toxicity in the sunflower plants at an optimal concentration of chromium (Cr50). The combined application of CeO_2_ nanoparticles and *S. aureus* efficiently ameliorated Cr-induced oxidative stress by promoting antioxidant enzymes activities and improved the photosynthetic activity by promoting chlorophyll contents in plants. In addition, CeO_2_ NPs and *S. aureus* decreased the Cr accumulation in roots and shoots in plants tolerating the highest chromium concentration (Cr50). The high concentration of chromium negatively affects morphological and agronomical parameters of plants through altering the water use efficiency, stomatal conductance, and photosynthesis process (Figures 1A–F, 2A–D; Ertani et al., 2017). In the present study, we evaluated that the application of CeO_2_ NPs alleviated the adverse impacts of chromium and improved plant agronomic characteristics (Figures 1A–F). Similar findings were reported by Gohari et al. (2020), where TiO_2_ NPs improved the agronomic traits in *Moldavian balm* under stress (Gohari et al., 2020). The large surface area and small size of the nanoparticles allow nanoparticles to penetrate in plant cells and then alleviate the negative effect of metal stress by improving plant growth (Singh et al., 2019). This increase in plant growth might be attributed by increases in water use efficiency, transpiration rate, stomatal conductance, and photosynthetic rate (Figures 2A–D; Hezaveh et al., 2019). The combined application of *S. aureus* and CeO_2_ NPs further improved the plant morphological and agronomic parameters as compared to the only application of NPs. It is well established that bacteria can increase essential nutrient availability to the plant (Ren et al., 2019). The bacteria also can degrade and transform the metals into less toxic forms and decrease the stress on the plant due to metal toxicity. Similarly, in this study, the application of *S. aureus* improved plant growth and biomass production due to its ability to detoxify the high concentration of chromium. The bacteria can improve plant nutrition through phosphorus solubilization, nitrogen fixation, and secretion of hormones and enzymes essential for plant growth and ultimately enhance the phytoremediation potential of plants (Marciano Marra et al., 2012; Matse et al., 2020).

The increased metal stress in various plant species resulted in a decrease in chlorophyll a, chlorophyll b, total chlorophyll, and carotenoid content, ultimately disrupting of whole photosynthesis process (Chandra and Kang, 2016; Sytar et al., 2019). The reduction in photosynthesis under metal stress is considered a sign of oxidative stress due to the rapid decrease in photosynthetic pigments and reduction in light absorbance capacity (Ashfaque et al., 2017). It was evident that the application of CeO_2_ NP improved the chlorophyll *a*, chlorophyll *b,* and total carotenoid content in plants under chromium stress and reduced the oxidative stress (Figures 3A–D). The beneficial role of many nanoparticles in enhancing the chlorophyll contents and improving the plant photosynthetic activity has been reported by many researchers. Such as application of CeO_2_ NPs acted as a catalyst in the production of *Chl a* and *Chl b* contents and increased the production of carotenoid contents in plant (Kataria et al., 2019; Etesami et al., 2021). Similarly, the foliar application of cerium oxide nanoparticles improved the photosynthesis pigment in *Calendula officinalis* (Jahani et al., 2019). The decline in antioxidant enzyme activity plays a crucial role in producing oxidative stress in plants suffering from chromium stress (Etesami, 2018). The significant increase in the chlorophyll contents and photosynthesis activity due to combined application of *S. aureus* and CeO_2_ NPs might be attributed to the ability of bacteria to increase the availability of micronutrients, decrease in bioavailability of chromium, and increase the ability of the plant to tolerate metal stress (Rizwan et al., 2019; Etesami et al., 2021). Similar to our findings, the application of bacteria improved the chlorophyll *a*, chlorophyll *b*, and carotenoid contents in the sunflower plant under Pb stress (Saleem et al., 2018). In another study, the TiO_2_ NPs improved the photosynthetic activity in soybean plants under Cd stress by entering the chloroplast and enhancing light adaption and electron transfer (Singh and Lee, 2016).

The increasing concentration of chromium declined the enzyme activity in the plants (Figures 4A–D). At the highest concentration of Cr50, the SOD activity, POD activity, CAT activity, and APX activity reduced sharply due to damage to the antioxidant enzymes metabolism induced by chromium toxicity. It is well reported that metal toxicity decreases the catalytic activities in the plant which ultimately decrease the activity of the antioxidant enzymes (Bhaduri and Fulekar, 2012). The production of ROS attacks antioxidants enzymes, consumes a huge concentration of antioxidant enzymes, and generates oxidative stress by disturbing the redox homeostasis in plants (He et al., 2017; Ahmad et al., 2019). This oxidative stress can cause severe damage to specific proteins and destruction to the cell structure and function (Ahmad et al., 2019). The application of nanoparticles has been reported to improve SOD, CAT, and POD activity without producing the hydroxyl radicals and improving the plants’ overall growth.

The chromium stress in plants leads to the production and accumulation of toxic substances such as reactive oxygen species (ROS) that initiate the lipid peroxidation and damage to cell structure (Figures 5A–C; Ghori et al., 2019; Jahani et al., 2019). The production of ROS initiates the death of plant cells by damaging specific proteins, lipids, and nucleic acids and decreases the chlorophyll contents (Sytar et al., 2019). This damage can be identified by increased production and accumulation of MDA, and increased concentration of electrolytes leakage (EL) and high content of H_2_O_2_ (Mallhi et al., 2019). Under metals stress, the concentrations of MDA, EL, and H_2_O_2_ increased in the plants (Berni et al., 2019; Ghori et al., 2019). The nanoparticle has been found to reduce ROS production in plants under metal stress. TiO_2_ NPs and Fe NPs have been found effective in reducing the abiotic stress in plants by reducing ROS production in *Zea mays* and grape, respectively (Mozafari and Ghaderi, 2018; Singh et al., 2021). In the current study, the increased production of antioxidants enzymes due to the positive effect of CeO_2_ NPs ultimately decreased the production of EL, MDA, and H_2_O_2_ contents in plants under chromium stress. In the plants, ROS production is initiated in response to stress, and antioxidants act as a defense mechanism against stress condition and act as a front line to cope with and protect the plant cell from free radicals and minimize the damage initiated by ROS in response to stress (Kohli et al., 2017). Antioxidant enzymes scavenge the H_2_O_2_ contents APX and SOD cope with ROS and neutralize the superoxidase radicals. Our study showed that CeO_2_ NPs increased the production of antioxidants enzymes, which resulted in decreased activity of EL, MDA, and H_2_O_2_ and alleviated the metal stress in the plant. Our findings agree with the aforementioned studies where the application of nanoparticles enhanced antioxidant enzyme activities in response to oxidative stress (Hussain et al., 2019b; Sun et al., 2019). The increased level of antioxidant enzyme activity might be linked to the antioxidant ability of nanoparticles, which enhances the plants’ activities (Jahani et al., 2019). It was found that the increasing concentration of Ag NPs increased the catalase and peroxidase activity in the *Lycopersicon esculentum* (Karami Mehrian et al., 2016). Similarly, wheat plants treated with silver and gold NPs significantly improved the plant dry biomass and improved the SOD, CAT, and APX activity in plants under abiotic stress (Manaf et al., 2021). The increase in antioxidant enzymes activity and decrease in ROS contents with the application of *S. aureus* may be attributed to bacteria’s ability to increase mRNA/gene expression of antioxidants in inoculated plants compared to non-inoculated plants (Gururani et al., 2013; Khan et al., 2022). The inoculation of bacteria enhanced the expression level of various ROS scavenging enzymes and increased the proline contents in potatoes plants under stress (Gururani et al., 2013).

The application of CeO_2_ nanoparticles reduces the uptake and accumulation of Cr^+6^ and Cr^+3^ in the sunflower root and shoots despite increasing chromium concentration (Figures 6A–D). Many studies reported that exogenous application of nanoparticles could reduce the uptake of toxic metals by plants (Hussain et al., 2019b; Rizwan et al., 2019). The use of TiO_2_ NPs in rice plants grown hydroponically reduced the concentration of Pb in roots and shoots and improved plant growth (Cai et al., 2017). Similarly, ZnO NPs decreased the arsenic concentration in the rice plat’s roots and leaves suffering from arsenic stress (Wu et al., 2020). It is well reported that nanoparticles can absorb and transform the heavy metals in the soils by reducing their mobility and bioavailability such as Fe_3_O_4_ NPs reduced the mobility of Cd in the soil and reduced its bioavailability to the plants (Chen et al., 2018; Hussain et al., 2019a). Thus, the decreased accumulation of chromium in the sunflower plant can be attributed to the ability of the nanoparticle to decrease the bioavailability of chromium. Further, most of the NPs accumulate in the cell wall, bind with the heavy metals, make them unavailable, and hinder the migration of heavy metals in the plant (Molnár et al., 2020). The bacteria can tolerate metal toxicity and break down and remove the heavy metal through their metabolic process. Bacteria can reduce the bioavailability of heavy metals through biosorption, bioaccumulation, biotransformation, bio-precipitation, and bio-crystallization in contaminated soil (Medfu Tarekegn et al., 2020). In this study, the application of bacteria transformed the Cr^+6^ into Cr^+3^ through their metabolic process. It reduced the bioavailability of the chromium to the plant, which ultimately reduced its bioaccumulation in plant roots and shoots. Similarly, the application of *S. aureus* in wheat plants reduced the uptake and accumulation of chromium in plant roots and shoots grown in chromium-contaminated soil (Zeng et al., 2020). The nanoparticles can improve bacterial growth when applied in small amounts; for example, the use of TiO_2_ NPs improved the performance of plant growth-promoting rhizospheric bacteria in plants under stress (Timmusk et al., 2018).

## Conclusion

The application of nanoparticles is a promising approach that has the tremendous potential to protect the plant from metal-induced stress. The CeO_2_ NPs improved the morphological, physiological, and biochemical properties and overall growth and biomass production of sunflower plants grown in the high chromium concentration. The CeO_2_ NPs reduce the H_2_O_2_ stress, enhance the antioxidant enzymatic activities, and ultimately alleviate plants’ oxidative stress due to chromium toxicity. Along with nanoparticles, bacteria also have a prominent role in alleviating the metal-induced toxicity through their metabolic process and reducing the bioavailability of Cr to the plant. The combined application of *S. aureus* and CeO_2_ nanoparticles improved the metabolic process, triggered the activation of the enzymatic defense system, and thus enhanced the plant performance in sunflower plants under chromium stress. The use of nanoparticles and bacteria in combination could be a novel way to clean up contaminated soil and strengthen plants to withstand metal-induced stress. The novel application of nanoparticles in agriculture may aid in meeting rising food demand while also ensuring environmental sustainability. However, there is still needed to further explore about action mechanism of nanoparticles, permissible limit and ecotoxicity in edible crops.

## Data Availability Statement

The raw data supporting the conclusions of this article will be made available by the authors, without undue reservation.

## Author Contributions

JM: conceptualization and project administration. HA: data curtain and investigation. MO: funding and resources allocation. YA: formal analysis and software. FC: methodology and data curtain. MA: formal analysis and project administration. AH: review and editing and formal analysis. SH: investigation and formal analysis. MS: writing an original draft, methodology, and review and editing. All authors contributed to the article and approved the submitted version.

## Funding

This work was supported by the National Natural Science Foundation of China (no. 51974313) and the key project of Jiangsu Key Laboratory of Coal-Based Greenhouse Gas Control and Utilization (2020ZDZZ03). The authors extend their appreciation to the Researchers Supporting Project number (RSP-2021/374) King Saud University, Riyadh, Saudi Arabia.

## Conflict of Interest

The authors declare that the research was conducted in the absence of any commercial or financial relationships that could be construed as a potential conflict of interest.

## Publisher’s Note

All claims expressed in this article are solely those of the authors and do not necessarily represent those of their affiliated organizations, or those of the publisher, the editors and the reviewers. Any product that may be evaluated in this article, or claim that may be made by its manufacturer, is not guaranteed or endorsed by the publisher.
